# Non-Alcoholic Fatty Liver Disease and the Risk of Diabetes Mellitus by Menopausal Status: A Nationwide Cohort Study

**DOI:** 10.3390/jpm12040546

**Published:** 2022-03-30

**Authors:** Jungeun Shin, Soo Jung Choi, Han Rim Lee, Kyungdo Han, Jiwon Chang, Su-Min Jeong, Yun-sun Choi, Daeho Lee, Dong Wook Shin

**Affiliations:** 1 Department of Family Medicine & Supportive Care Center, Samsung Medical Center, Sungkyunkwan University School of Medicine, Seoul 06351, Korea; lovevsv@gmail.com (J.S.); lhl1221@gmail.com (H.R.L.); smjeong.fm@gmail.com (S.-M.J.); 2 International Healthcare Center, Samsung Medical Center, Seoul 06351, Korea; 3 Department of Family Medicine, Gachon University Gil Medical Center, Incheon 21565, Korea; 4 Department of Statistics and Actuarial Science, Soongsil University, Seoul 06978, Korea; hkd917@naver.com; 5 Total Healthcare Center, Kangbuk Samsung Hospital, Sungkyunkwan University School of Medicine, Seoul 04514, Korea; wldnjs5353@gmail.com; 6 Samsung Medical Center, Department of Obstetrics and Gynecology, Sungkyunkwan University, Seoul 06351, Korea; ys1102.choi@samsung.com; 7 Division of Endocrinology, Department of Internal Medicine, Gil University Hospital, Incheon 21565, Korea; drhormone@naver.com; 8 Department of Clinical Research Design and Evaluation, SAIHST, Sungkyunkwan University, Seoul 06351, Korea; 9 Department of Digital Health, SAIHST, Sungkyunkwan University, Seoul 06351, Korea

**Keywords:** diabetes mellitus, nonalcoholic fatty liver, menopause

## Abstract

Background: Non-alcoholic fatty liver disease (NAFLD) is regarded as a risk factor for type 2 diabetes mellitus (DM). Menopausal status also influences T2DM risk, where estrogen is presumed to play a protective role by decreasing insulin resistance. As such, we investigated the association between NAFLD and DM risk according to menopausal status. Objectives: We sought to examine the association between NAFLD and DM incidence in pre- and post-menopausal women. Methods: A total of 842,772 pre-menopausal and 1,074,767 post-menopausal women who underwent health examinations between 2009 and 2014 were included from the Korean National Health Insurance Service database. Multivariate Cox proportional hazard analyses were performed to evaluate the association between the risk of DM according to menopausal status and NAFLD, defined by a fatty liver index >60. Results: During the mean follow-up period (7.8 years), DM was diagnosed in 33,461 (4.0%) of pre-menopausal women and 121,102 (9.4%) post-menopausal women. A stronger association between NAFLD and the risk of DM was found in pre-menopausal women (multivariable-adjusted hazard ratio [aHR], 3.60; 95% confidence interval [CI], 3.48–3.71) than in post-menopausal women (aHR, 2.24; 95% CI, 2.21–2.28) (*P*-interaction < 0.01). Subgroup analyses among women aged 45 to 55 years also showed a stronger association in pre-menopausal (aHR, 3.35; 95% CI, 3.21–3.49) than in post-menopausal women (aHR, 2.83; 95% CI, 2.68–2.98) (*P*-interaction < 0.01). Conclusions: The association between NAFLD and DM was stronger in pre-menopausal women than in post-menopausal women. This might be due to the protective effect of estrogen, which is possibly in higher production in the peripheral fat tissue of post-menopausal women with NAFLD.

## 1. Introduction

The prevalence of type 2 diabetes (DM) increased from 9.6% in 2007–2009 to 10.8% in 2016–2017 in adults over 30 years of age, and it affected approximately 5 million Korean adults in 2016 [1]. DM is associated with numerous medical complications and shortened life expectancy [2]. Many previous studies have attempted to find modifiable risk factors to control the disease [1]. One of the risk factors for DM is Non-Alcoholic Fatty Liver Disease (NAFLD) [3]. The mechanism of the association is not fully understood, but insulin resistance is presumably involved [4]. Previous studies have suggested NAFLD as a precise early predictor of DM incidence, indicating that hepatic steatosis observed in NAFLD may be the cause of insulin resistance [5,6].

In previous studies, menopausal status has been suggested as a potential risk factor for NAFLD, as the prevalence increased gradually among women aged 20–49 years and spiked suddenly once age exceeded 50 years [7]. Menopause is also known to be a risk factor for DM, independent of age and high BMI [8]. Estrogen plays a role in slowing the progression of chronic liver diseases and insulin resistance by suppressing inflammation, but changes in estrogen levels following menopause result in an increased risk of DM [9]. It has been shown that the prevalence of NAFLD and DM are higher in post-menopausal women than in pre-menopausal women.

Although many previous studies have investigated the relationship between NAFLD and DM, and it is known that both NAFLD and DM are affected by menopausal changes, the relationship between NAFLD and DM in pre- and post-menopausal women has not been thoroughly studied. Thus, we examined the association between NAFLD and DM in pre-menopausal and post-menopausal groups in this study. In order to minimize the influence of age-related factors, we examined the association between NAFLD and DM in pre-menopausal and post-menopausal groups aged 45 to 54 years, where pre-menopausal and post-menopausal women co-exist.

## 2. Materials and Methods

### 2.1. Data Source and Study Setting

In South Korea, the National Health Insurance Service (NHIS) is a single insurer that provides mandatory universal coverage for 97% of the Korean population and administers a program to provide medical aid for the remainder of the population.

For screening purposes, the NHIS operates a national health checkup program for the general Korean population, which includes cardiovascular health screening for everyone over 40 years old and all who are employed regardless of age. The NHIS also operates the National Cancer Screening Program (NCSP) for all individuals according to age. For example, breast cancer screening is indicated for women aged 40 years and older. Therefore, the NHIS has a comprehensive demographics database, including age, sex, socio-economic variables, type of eligibility, income level, etc., and a medical treatment database comprised of medical providers’ service claims. The health examination database also includes lifestyle and daily performance information from self-administered questionnaires.

### 2.2. Study Population

Data corresponding to women over forty who underwent both cardiovascular and breast cancer screening from 1 January 2009 to 31 December 2014 were collected from the NHIS database. The first screening date was set as the baseline for those who had two or more screenings.

**Figure 1:**
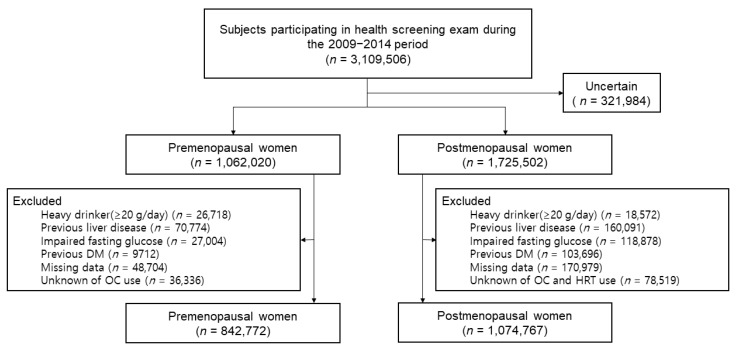
Flow chart of the study population.

Among 3,109,506 women who were enrolled, we excluded those whose information regarding menopausal status was uncertain (*n* = 321,984). In addition, those who were considered heavy drinkers with more than 20g of alcohol consumption per day (*n* = 26,718 pre-menopausal, *n* = 18,572 post-menopausal); those who were diagnosed with liver disease, including hepatocellular carcinoma, liver cirrhosis, and other chronic liver diseases (*n* = 70,774 pre-menopausal, *n* = 160,091 post-menopausal); those with a fasting glucose level of more than 126 mg/dL (*n* = 27,004 pre-menopausal, *n* = 118,878 post-menopausal); those who had already been diagnosed with DM (*n* = 9712 pre-menopausal, *n* = 103,696 post-menopausal); and those who answered ‘unknown’ to the questionnaires about both hormone replacement therapy and oral contraceptive history (*n* = 170,979 post-menopausal) or oral contraceptive history alone (*n* = 36,336 pre-menopausal) were excluded. As a result, 1,917,539 total individuals (842,772 pre-menopausal and 1,074,767 post-menopausal women) were included in the analysis (Figure 1).

### 2.3. Data Collection

Participants answered a self-administered questionnaire about health-related behaviors and menstrual and reproductive history. Data on age of menarche were divided into two groups by age (≤13 years or ≥13 years), and data on age at menopause were divided into five groups by age (<40 years, 40–44 years, 45–49 years, 50–54 years, or ≥55 years).

Participants’ birth-related histories were also collected, including parity (0, 1, or ≥2 children), total lifetime breastfeeding history (never, <6 months, 6–12 months, or ≥12 months), the duration of hormone replacement therapy (HRT) (never, <2 years, 2–5 years, ≥5 years, and unknown), and the duration of oral contraceptive use (never, <1 year, ≥1 year, and unknown).

Body mass index (BMI) was calculated by dividing weight (kg) by height in meters squared (m^2^) [10,11], and waist circumstance (WC) was measured at the middle of the lower margin of the final palpable rib and the top of the iliac crest [12].

Participants were also categorized into three groups by smoking status, including never smokers, former smokers, and current smokers, and further categorized by alcohol consumption into never drinkers and current drinkers (<20 g/day). Regular physical activity was defined as more than 30 min and more than 5 days per week of moderate physical activity over the past week. The presence of hypertension, dyslipidemia, chronic kidney disease, and familial history of stroke and heart disease was determined by the diagnosis of a physician, prescription history, or self-reporting.

### 2.4. Non-Alcoholic Fatty Liver Disease Evaluation

NAFLD was assessed according to the fatty liver index (FLI), which is a useful non-invasive surrogate predictor of NAFLD, by using an algorithm based on BMI, WC, triglyceride (TG), and gamma glutamyl transferase (GGT), where FLI = (e^0.953 × ln(TG)^ + 0.139 × BMI + 0.718 × ln(GGT) + 0.053 × WC − 15.745)/(1 + e^0.953 × ln(TG) + 0.139 × BMI + 0.718^ × ln(GGT) + 0.053 ^× WC − 15.745)^ × 100 [13]. FLI varies from 0 to 100, where FLI < 30 (negative likelihood ratio = 0.2) rules out fatty liver disease (FLD) and FLI ≥ 60 (positive likelihood ratio = 4.3) rules in FLD [14]. Compared to ultrasonography or liver biopsy, which are the conventional diagnostic methods for NAFLD, FLI shows 84% accuracy for predicting NAFLD [15], thus enabling us to identify NAFLD in large-scale epidemiological research cohorts [16]. 

### 2.5. Study Outcomes and Follow-Up

Newly diagnosed DM was determined by the presence of the ICD-10 ofHE11 diagnostic codes in the medical treatment database or the prescription of a hypoglycemic agent.

The cohort was followed from the primary health checkup date to the date of DM diagnosis, death, or the study endpoint (31 December 2017). The mean (standard deviation) follow-up duration was 7.8 years (1.5), and the maximum was 9.0 years.

### 2.6. Statistical Analysis

Descriptive analysis was made with the data at the time of participants’ enrollment. Continuous variables are shown as mean ± standard deviation, and categorical variables are represented as numbers and percentages. DM incidence rates were measured by dividing the number of incident cases by the total follow-up period.

Several multivariable-adjusted Cox proportional hazard models were applied: (1) Model 1 was non-adjusted; (2) Model 2 was adjusted for income, smoking status, alcohol consumption, level of physical activity, and fasting glucose level; (3) Model 3 was further adjusted for comorbidities, such as hypertension, dyslipidemia, and chronic kidney disease; (4) Model 4 was further adjusted for reproductive factors including the age at menarche, parity, duration of breastfeeding, duration of oral contraceptive use in pre-menopausal women, and additionally for age at menopause and the duration of HRT in post-menopausal women.

Considering the relationship between NAFLD and DM risk can be affected by specific age-related factors, stratification analysis was performed by age group (40–49 years, 50–59 years, and ≥60 years), and additional analyses were performed in a narrower age range of 45 to 54 years, in which the perimenopausal transition occurs [17].

SAS version 9.4 (SAS Institute Inc., Cary, NC, USA) was used to perform statistical analyses. *p*-values < 0.05 were considered statistically significant.

### 2.7. Ethics Statement

This study was approved by the Institutional Review Board (IRB) of Samsung Medical Center (IRB No. 2019-07-045). The need for informed consent was relinquished by the IRB.

## 3. Results

### 3.1. Baseline Characteristics of the Study Participants

The basic characteristics of pre-menopausal and post-menopausal participants are described in Table 1 and reproduction-associated characteristics are in Table 2, stratified by DM status. In the pre-menopausal group, the prevalence of abdominal obesity was 9.78% and 32.17% in patients with and without DM, respectively. The mean FLI of the non-DM participants was 8.27, whereas that of the DM participants was 23.26. In the post-menopausal group, the prevalence of obesity was 23.58% in the non-DM group and 42.6% in the DM group. The mean FLI in non-DM participants was 15, whereas that of the DM participants was 27.1.

### 3.2. Associations between FLI and Type 2 DM Risk by Menopausal Status

During a mean follow-up of 7.8 years, 33,461 pre-menopausal women and 107,080 post-menopausal women were diagnosed with type 2 DM.

**Figure 2:**
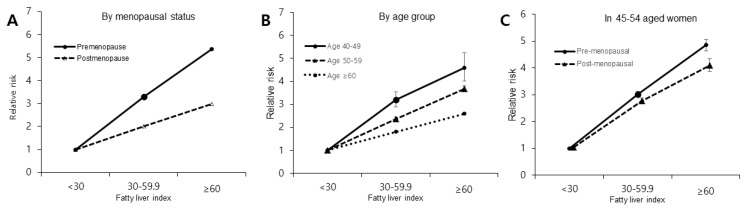
HR and 95% Cl of type 2 diabetes mellitus incidence according to fatty liver index. (**A**) associations between FLI and type 2 DM risk by menopausal status. (**B**) associations between FLI and type 2 DM risk by age group. (**C**) associations between FLI and type 2 DM risk by menopausal status in women between the ages of 45 and 54 years old.

FLI has a stronger relationship with the occurrence of DM in pre-menopausal women than in post-menopausal women. The adjusted HR (aHR) for DM was 3.60 (95% confidence interval [CI], 3.48–3.71) for FLI ≥ 60 compared to FLI < 60, whereas the aHR of post-menopausal women was 2.24 (95% CI, 2.21–2.28; *p* trend < 0.001). Also, when separating FLI into three groups, the aHR (95% CI) for DM in pre-menopausal women with FLI ≥ 60 was 5.37 (5.19–5.55), and that for pre-menopausal women with FLI < 60 was 3.29 (3.21–3.38) compared to FLI < 30. In post-menopausal women, the aHR (95% CI) for DM was 2.98 (2.92–3.03) for FLI ≥ 60 and 2.01 (1.99–2.04) for FLI < 60 compared to FLI < 30 (*p* trend < 0.001), as described in Table 3 and Figure 2A.

### 3.3. Associations between FLI and Type 2 DM Risk by Age Group

The younger age group, 40–49 years of age, showed the strongest association between FLI and DM occurrence (Figure 2B). When dividing FLI into three age groups, the HR (95% CI) for DM in the age group between 40–49 years of age was 4.58 (4.02–5.26) for FLI ≥ 60 and 3.21 (2.90–3.55) for FLI < 60 compared to FLI < 30 (*p* trend < 0.001), whereas in the 50–59-year age group, the HR was 3.68 (3.57–3.80) for FLI ≥ 60 and 2.37 (2.31–2.42) for FLI < 60 compared to FLI < 30. In those older than 60, the HR for DM was 1.81 (1.78–1.84) for FLI < 60 and 2.59 (2.54–2.65) for FLI ≥ 60 compared to FLI < 30 (*p* trend < 0.001), as described in Table 4.

### 3.4. Associations between FLI and Type 2 DM Risk by Menopausal Status in Women between the Ages of 45 and 54

To eliminate the effect of age-related factors on the association between NAFLD and DM risk, we performed additional analyses in a narrow age range (45–54-year-olds). In this age group, pre-menopausal status was associated with the occurrence of DM even after controlling for age (Figure 2C).

In the pre-menopausal group, the HR (95% CI) for DM was 3.35 (3.21–3.49) for FLI ≥ 60 compared to FLI < 60. Moreover, the HR (95% CI) for DM was 3.02 (2.93–3.12) for FLI < 60 and 4.85 (4.64–5.07) for FLI ≥ 60 compared to FLI < 30 (*p* trend < 0.001). In the post-menopausal group, the HR (95% CI) for DM was 2.83 (2.68–2.98) for FLI ≥ 60 compared to FLI < 60. Compared to those with FLI < 30, the HR (95% CI) for DM was 2.72 (2.61–2.84) for FLI < 60 and 4.06 (3.84–4.30) for FLI ≥ 60 (*p* trend < 0.001), as depicted in Table 5.

## 4. Discussion

In this cohort study of 1,917,539 Korean women, we found that the presence of NAFLD was associated with a higher risk of developing DM in pre-menopausal women than in post-menopausal women. Even in a narrower age spectrum of 45–54 years, where the transition to menopause is most common, women in the pre-menopausal state with NAFLD had a higher risk of DM than those in the post-menopausal state.

In menopause, various hormonal changes occur [18], including a reduction in estrogen levels, leading to an androgen-dominated metabolic environment [19]. Changes in the androgen-to-estrogen ratio after menopause appear to be accompanied by an increase in intra-abdominal fat deposition; this increase in visceral adiposity during the menopausal transition is thought to be associated with worsening insulin resistance [17,20]. Furthermore, an increase in insulin resistance results in not only elevated free fatty acid levels and metabolic syndrome, but also NAFLD. Additionally, a previous study suggested that the hypoestrogenic period accompanying menopause facilitates the onset of NAFLD and its progression [9]. As a result, the risk of DM increases due to altered insulin resistance, which is associated with hormonal changes during the menopausal transition and the hepatic steatosis of NAFLD.

In this large-scale cohort study, the association between NAFLD and DM was more obvious in pre-menopausal than post-menopausal women. Hormonal changes in the peripheral tissue during menopause may explain the protective effect from DM seen in post-menopausal NAFLD women. In post-menopausal women, although the ovarian release of estradiol decreases, estrogens are synthesized peripherally in adipose tissue by androgen aromatization [21], while obesity has a direct inhibitory effect on estradiol production from the ovaries in pre-menopausal women [22,23,24]. Thus, while menopause itself would lead to visceral obesity and insulin resistance, increased estrogen production in post-menopausal women with visceral obesity would attenuate the association between NAFLD and the development of DM compared to that in the pre-menopausal women.

Many studies suggest that the changes in insulin resistance associated with NAFLD play a key role in the development of DM [16,25]. Insulin resistance in pre-menopausal women with NAFLD, even when under the protection of estrogen, may lead to the higher risk of DM seen in this study. As a result, increased insulin resistance caused by pre-menopausal NAFLD seems to result in DM more than NAFLD in a hypoestrogenic environment, despite the protection of estrogen.

The analysis was performed in women aged 45 to 54, where pre- and post-menopausal women co-exist, to minimize the impact of age-related factors. After adjusting for age and other confounding factors, the strength of the association between NAFLD and DM risk in pre-menopausal women versus post-menopausal women decreased; however, there was still a significant difference between the two. This result supports the main results of our study and the above explanations that peripherally originated estrogen could have a protective effect against DM in obese post-menopausal women.

It is important to recognize that pre-menopausal women with NAFLD may be at increased risk for developing DM. As the prevalence of NAFLD in the younger population is increasing [26], it is important for clinicians to make an effort to screen for DM and reduce risk factors for DM in pre-menopausal women with NAFLD. Second, menopausal hormone replacement therapy may potentially benefit the mitigation of DM risk, especially in post-menopausal women without NALFD. While menopausal hormonal therapy (MHT) has not been approved by the FDA for the prevention of type 2 DM, it has been suggested that MHT using estrogens delays the onset of type 2 DM [27]. Therefore, DM risk reduction by MHT should be considered, especially for women without NALFD.

Several limitations exist in this study. First, the existence of fatty liver was only measured by the FLI. Since FLI has an accuracy of 0.84 (95% CI, 0.81–0.87) in detecting NAFLD, there may have been participants with unidentified NAFLD that could have been detected by ultrasonographic evaluation or liver biopsy. However, FLI is a low-cost and convenient method that only requires a blood test and is commonly performed in routine medical visits. Moreover, FLI is also highly predictive of DM development, especially in younger people [28]. Second, this study mainly included people who had health examinations, so it is expected that the participants had a higher possibility of leading a healthier lifestyle than the general population. Third, we determined the study groups from data when the participants were enrolled, so changes during the follow-up periods were not reflected. Fourth, as we collected participants’ menopausal information through a self-administered questionnaire, misclassifications could have been made. Fifth, due to the observational nature of this study, effects caused by age and menopause could not be fully distinguished. Sixth, even though known confounders were adjusted for, unmeasured confounding factors may exist. Seventh, the level of insulin and estrogen were not directly measured in this study, so the direct relationship of estrogen and insulin resistance were not included. Despite these limitations, this study was the first to use a large cohort to evaluate the association between NAFLD and DM incidence in pre- and post-menopausal women to our knowledge.

## 5. Conclusions

In conclusion, this study showed that the association between NAFLD and DM incidence is stronger in pre-menopausal women than in post-menopausal women in the Korean population. Further studies are needed to better understand the mechanism of this association with insulin level measurement and to find effective measures to control disease progression in this subgroup.

## Figures and Tables

**Table 1:** Baseline Characteristics and comorbidity of the Study Population according to Menopausal Status.

	Premenopausal			Postmenopausal		
	DM			DM		
	No	Yes	*p*-Value	No	Yes	*p*-Value
	*n* = 809,311	*n* = 33,461		*n* = 1,072,779	*n* = 121,102	
	*n* (%)	*n* (%)		*n* (%)	*n* (%)	
Age, years, mean (SD)	44.9 (±3.9)	46.5 (±4.1)	<0.001	61.1 (±8.3)	63.2 (±8.0)	<0.001
Body mass index, kg/m^2^, mean (SD)	23.0 (±2.9)	25.8 (±3.6)	<0.001	23.9 (±3.0)	25.43 (±3.3)	<0.001
<18.5	24,048 (3.0)	203 (0.6)	<0.001	23,587 (2.4)	1187 (1.1)	<0.001
18.5–22.9	416,750 (51.5)	7056 (21.1)		362,309 (37.4)	22,050 (20.6)	
23–24.9	189,034 (23.4)	7660 (22.9)		259,595 (26.8)	26,176 (24.5)	
25–29.9	160,750 (19.9)	14,378 (43.0)		291,969 (30.2)	48,423 (45.2)	
≥30	18,729 (2.3)	4164 (12.4)		30,227 (3.1)	9244 (8.6)	
Abdominal obesity, * *n* (%)	79,185 (9.8)	10,764 (32.2)	<0.001	228,216 (23.6)	45,615 (42.6)	<0.001
Smoking status, *n* (%)			<0.001			<0.001
Never	775,405 (95.1)	31,749 (94.9)		936,051 (96.7)	102,347 (95.6)	
Ex-smoker	11,946 (1.5)	455 (1.4)		9307 (1.0)	1204 (1.1)	
Current	21,960 (2.7)	1257 (3.8)		22,329 (2.3)	3529(3.3)	
Regular alcohol consumption, *n* (%)	25,648 (76.7)	592,085 (73.2)	<0.001	96,092(89.7)	850,544 (87.9)	<0.001
Regular physical activity, *n* (%)	139,015 (17.2)	5645 (16.9)	0.145	177,369(18.3)	18,711 (17.5)	<0.001
Income, *n* (%)			<0.001			0.018
1st quartile (lowest)	207,656 (25.7)	9051 (27.1)		221,270 (22.9)	24,639 (23.0)	
2nd quartile	162,209 (20.0)	7394 (22.1)		179,907 (18.6)	19,970 (18.7)	
3rd quartile	177,766 (22.0)	7518 (22.5)		238,101 (24.6)	26,633 (24.9)	
4th quartile (highest)	261,680 (32.3)	9498 (28.4)		328,409 (33.9)	35,838 (33.5)	
Comorbid conditions, *n* (%)						
Hypertension	98,313 (12.2)	10,593 (31.7)	<0.001	392,104 (40.5)	64,101 (59.9)	<0.001
Hyperlipidemia	78,365 (9.7)	7837 (23.4)	<0.001	289,736 (29.9)	46,345 (43.3)	<0.001
Chronic kidney disease	33,282 (4.1)	1496 (4.5)	0.012	102,478 (10.6)	14,263 (13.3)	<0.001
History of stroke	1821 (0.5)	109 (0.6)	0.02	11,236 (1.8)	1850 (2.4)	<0.001
History of heart disease	3408 (0.9)	301 (1.6)	<0.001	30,001 (4.8)	5,845 (7.5)	<0.001
Systolic blood pressure, mmHg, mean (SD)	116.7 (±14.1)	123.89 (±15.6)	<0.001	124.7 (±16.0)	129.01 (±16.1)	<0.001
Diastolic blood pressure, mmHg, mean (SD)	72.85 (±9.9)	77.37 (±10.5)	<0.001	76.6 (±10.1)	78.61 (±10.1)	<0.001
Fasting glucose, mg/dL, mean (SD)	91.0 (±10.0)	101.4 (±12.4)	<.001	92.9 (±10.6)	101.35 (±12.4)	<0.001
Total cholesterol, mg/dL, mean (SD)	191.3 (±38.4)	205.0 (±39.7)	<0.001	209.0 (±43.2)	213.9 (±46.4)	<0.001
High density lipoprotein, mg/dL, mean (SD)	60.6 (±35.2)	55.6 (±32.3)	<0.001	58.4 (±35.7)	56.0 (±35.8)	<0.001
Low density lipoprotein, mg/dL, mean (SD)	114.4 (±70.4)	123.7 (±60.2)	<0.001	128.5 (±67.8)	130.2 (±80.3)	<0.001
AST **, mg/dL, mean (SD)	20.2 (20.1–20.2)	22.5 (22.4–22.6)	<0.001	23.5 (23.5–23.5)	24.9 (24.9–25.0)	<0.001
ALT **, mg/dL, mean (SD)	16.2 (16.1–16.2)	21.8 (21.7–22.0)	<0.001	19.3 (19.3–19.3)	22.8 (22.7–22.9)	<0.001
rGTP **, mg/dL, mean (SD)	16.5 (16.5–16.6)	23.7 (23.6–23.9)	<0.001	19.4 (19.3–19.4)	24.7 (24.6–24.8)	<0.001
Triglyceride ***, mg/dL, mean (SD)	86.0 (85.9–86.1)	125.4 (124.7–126.1)	<0.001	111.4 (111.3–111.5)	137.66 (137.2–138.1)	
Fatty liver index, *** mean (SD)	8.3 (8.3–8.3)	23.3 (23.0–23.5)	<0.001	15.0 (15.0–15.0)	27.1 (27.0–27.2)	<0.001

* Abdominal obesity: (Waist circumference ≥ 90 cm). ** AST: aspartate aminotransferase, ALT: alanine aminotransferase, GGT: gamma-glutamyl transferase. *** Fatty Liver Index = (e0.953 × loge (triglycerides) + 0.139 × BMI + 0.718 × loge (GGT) + 0.053 × waist circumference − 15.745)/(1 + e0.953 × loge (triglycerides) + 0.139 × BMI + 0.718 × loge (GGT) + 0.053 × waist circumference − 15.745) × 100.

**Table 2:** Baseline Characteristics related to reproduction of the Study Population according to Menopausal Status.

	Premenopausal	Postmenopausal		Premenopausal	Postmenopausal	
	DM			DM		
	No	Yes		No	Yes	
	*n* = 809,311	*n* = 33,461		*n* = 809,311	*n* = 33,461	
	*n* (%)	*n* (%)		*n* (%)	*n* (%)	
Age at menarche, mean (SD)	15.1 (±1.7)	15.3 (±1.8)	<0.001	16.4 (±1.8)	16.6 (±1.8)	<0.001
≤12	35,822 (4.4)	1511 (4.5)		9522 (1.0)	967 (0.9)	
13–14	25,8971 (32)	9542 (28.5)		120,916 (12.5)	11,529 (10.8)	
15–16	371,470 (45.9)	14,923 (44.6)		376,514 (38.9)	39,924 (37.3)	
>16	143,048 (17.7)	7485 (22.4)		460,735 (47.6)	54,660 (51.1)	
Age at menopause, mean (SD)				50.0 ± 4.0	50.1 ± 4.2	<0.001
<40				16,203 (1.7)	2156 (2.0)	<0.001
40–44				55,379 (5.7)	6521 (6.1)	
45–49				267,177 (27.6)	28,050 (26.2)	
50–54				530,168 (54.8)	57,067 (53.3)	
≥55				98,760 (10.2)	13,286 (12.4)	
Parity			<0.001			<0.001
1	32,668 (4.0)	1106 (3.3)		59,799 (6.2)	5121 (4.8)	
≥2	106,107 (13.1)	4056 (12.1)		884,637 (91.4)	99,855 (93.3)	
Nullipara	670,536 (82.9)	28,299 (84.6)		23,251 (2.4)	2104 (2.0)	
Duration of BF, months			<0.001			<0.001
<6	143,529 (17.7)	5609 (16.8)		65,555 (6.8)	5047 (4.7)	
6–12	202,041 (25.0)	6244 (18.7)		170,274 (17.6)	15,985 (14.9)	
≥12	215,666 (26.7)	8549 (25.6)		667,819 (69.0)	80,295 (75.0)	
Never	248,075 (30.7)	13,059 (39.0)		64,039 (6.6)	5753 (5.4)	
Total reproductive year, mean (SD)				33.6 ± 4.4	33.5 ± 4.6	<0.001
<30				133,153 (13.8)	16,011 (15.0)	<0.001
<35				405,523 (41.9)	44,041 (41.1)	
<40				369,681 (38.2)	39,277 (36.7)	
≥40				59,330 (6.1)	7751 (7.2)	
Duration of OC use, years			<0.001			<0.001
Never	705,694 (87.2)	28,573 (85.4)		818,371 (84.6)	89,583 (83.7)	
<1	77,285 (9.6)	3422 (10.2)		91,209 (9.4)	10,071 (9.4)	
≥1	26,332 (3.3)	1466 (4.4)		58,107 (6)	7426 (7.0)	
Duration of HRT, years						<0.001
Never				808,748 (83.6)	91,463 (85.4)	
<2				92,482 (9.6)	8977 (8.4)	
2–5				38,216 (4.0)	3542 (3.3)	
≥5				28,241 (2.9)	3098 (2.9)	

**Table 3:** Associations between fatty liver index and type 2 diabetes mellitus risk by menopausal status.

	FLI	Subjects (*n*)	Event (*n*)	Person Years	IR *	HR (95% CI)			
						Model 1 ^§^	Model 2	Model 3 ^¥^	Model 4 ^₮^
Pre-menopausal	<60	821,818	28,575	6,746,918.8	4.24	1 (Ref.)	1 (Ref.)	1 (Ref.)	1 (Ref.)
	≥60	20,954	4886	156,094.1	31.30	7.59 (7.36, 7.82)	4.58 (4.44, 4.73)	3.60 (3.48, 3.71)	3.55 (3.43, 3.66)
	<30	741,027	18,536	6,109,038.6	3.03	1 (Ref.)	1 (Ref.)	1 (Ref.)	1 (Ref.)
	<60	80,791	10,039	637,880.2	15.74	5.25 (5.12, 5.38)	3.66 (3.57, 3.75)	3.29 (3.21, 3.38)	3.26 (3.18, 3.34)
	≥60	20,954	4886	156,094.1	31.30	10.61 (10.28, 10.95)	6.45 (6.24, 6.66)	5.37 (5.19, 5.55)	5.30 (5.13, 5.49)
	*p* **	-	-	-	-	<0.001	<0.001	<0.001	<0.001
Post-menopausal	<60	1014,707	90,996	8,059,055.6	11.29	1 (Ref.)	1 (Ref.)	1 (Ref.)	1 (Ref.)
	≥60	60,060	16,084	428,743.0	37.51	3.35 (3.29, 3.40)	2.55 (2.50, 2.59)	2.24 (2.21, 2.28)	2.24 (2.20, 2.27)
	<30	780,961	52,147	6,275,573.4	8.31	1 (Ref.)	1(Ref.)	1 (Ref.)	1(Ref.)
	<60	233,746	38,849	1,783,482.2	21.78	2.63 (2.60, 2.67)	2.18 (2.15, 2.21)	2.01 (1.99, 2.04)	2.01 (1.98, 2.04)
	≥60	60,060	16,084	428,743.0	37.51	4.55 (4.47, 4.63)	3.36 (3.30, 3.42)	2.98 (2.92, 3.03)	2.97 (2.92, 3.03)
	*p* **	-	-	-	-	<0.001	<0.001	<0.001	<0.001

IR *: Incident rate (per 1000 person years); *p* **: *p* for trend; ^§^ Model 1: not adjusted; Model 2: adjusted for age, income, smoking status, alcohol consumption, level of physical activity, and fasting glucose level. ^¥^ Model 3: further adjusted for hypertension, dyslipidemia, and chronic kidney disease. ^₮^ Model 4: further adjusted for the duration of oral contraceptive use, parity, total duration of breast feeding, and age of menarche.

**Table 4:** Associations between fatty liver index and type 2 diabetes mellitus risk according to age.

	FLI	Subjects (N)	Event (n)	Person Years	IR *	HR (95% CI)		
						Model 1 ^§^	Model 2	Model 3 ^¥^
Age 40–49 years	<60	733,923	22,909	6,030,022.4	3.80	1 (Ref.)	1 (Ref.)	1(Ref.)
	≥60	17,731	3914	133,001.5	29.43	7.96 (7.69, 8.23)	3.84 (3.41, 4.34)	3.05 (2.70, 3.46)
	<30	666,609	15,027	5,497,107.7	2.73	1 (Ref.)	1(Ref.)	1(Ref.)
	<60	67,314	7882	532,914.8	14.79	5.48 (5.33, 5.63)	3.59(3.25, 3.96)	3.21 (2.90, 3.55)
	≥60	17,731	3914	133,001.5	29.43	11.08 (10.70, 11.48)	5.536 (4.88, 6.28)	4.58 (4.02, 5.26)
	*p* for trend					<0.001	<0.0001	<0.0001
Age 50–59 years	<60	554,894	36,393	4,507,739.1	8.07	1 (Ref.)	1 (Ref.)	1(Ref.)
	≥60	25,919	6934	188,469.5	36.79	4.63 (4.51, 4.75)	3.10 (3.01, 3.19)	2.63(2.55, 2.71)
	<30	454,837	21,428	3,727,324.9	5.75	1 (Ref.)	1 (Ref.)	1(Ref.)
	<60	100,057	14,965	780,414.2	19.18	3.36 (3.29, 3.43)	2.59 (2.53, 2.65)	2.37(2.31, 2.42)
	≥60	25,919	6934	188,469.5	36.79	6.52 (6.34, 6.70)	4.28 (4.15, 4.41)	3.68(3.57, 3.80)
	*p* for trend						<0.0001	<0.0001
Age ≥60 years	<60	547,708	60,269	4,268,212.9	14.12	1 (Ref.)	1 (Ref.)	1(Ref.)
	≥60	37,364	10,122	263,366.2	38.43	2.73 (2.67, 2.79)	2.25 (2.20, 2.29)	2.03 (2.99, 2.08)
	<30	400,542	34,228	3,160,179.5	10.83	1 (Ref.)	1 (Ref.)	1(Ref.)
	<60	147,166	26,041	1,108,033.4	23.50	2.17 (2.14, 2.21)	1.93 (1.9, 1.96)	1.81 (1.78, 1.84)
	≥60	37,364	10,122	263,366.2	38.43	3.56 (3.48, 3.64)	2.87 (2.80, 2.93)	2.59 (2.54, 2.65)
	*p* for trend					<0.001	<0.0001	<0.0001

IR *: Incident rate (per 1000 person years); ^§^ Model 1: not adjusted; Model 2: adjusted for age, income, smoking status, alcohol intake, level of physical activity, and fasting glucose level; ^¥^ Model 3: further adjusted for hypertension, dyslipidemia, and chronic kidney disease.

**Table 5:** Associations between fatty liver index and type 2 diabetes risk by menopausal status in 45-54-year-old women.

		Subjects (*n*)	Event (*n*)	Person Years	IR *	HR (95% CI)			
						Model 1 ^§^	Model 2	Model 3 ^¥^	Model 4 ^₮^
	Menopause								
	No	392,234	20,037	3,205,321.1	6.3	1 (Ref.)	1 (Ref.)	1 (Ref.)	
	Yes	185,299	11,213	1,505,725.6	7.4	1.19 (1.17, 1.22)	0.95 (0.92, 0.97)	0.91 (0.89, 0.93)	
Pre-menopause	FLI								
	<60	381,736	17,293	3,128,111.4	5.5	1 (Ref.)	1 (Ref.)	1 (Ref.)	1 (Ref.)
	≥60	10,498	2744	77,209.7	35.5	6.60 (6.34, 6.87)	4.27 (4.10, 4.45)	3.35 (3.21, 3.49)	3.30 (3.16, 3.44)
	<30	338,630	11,243	2,789,697.0	4.0	1 (Ref.)	1 (Ref.)	1 (Ref.)	1 (Ref.)
	<60	43,106	6050	338,414.4	17.9	4.49 (4.35, 4.63)	3.39 (3.29, 3.50)	3.02 (2.93, 3.12)	3.00 (2.90, 3.10)
	≥60	10,498	2744	77,209.7	35.5	9.07 (8.70, 9.46)	5.89 (5.64, 6.14)	4.85 (4.64, 5.07)	4.79 (4.58, 5.01)
	*p* for trend					<0.0001	<0.0001	<0.0001	<0.0001
Post- menopause	FLI								
	<60	178,301	9502	1,454,328.6	6.5	1 (Ref.)	1 (Ref.)	1 (Ref.)	1 (Ref.)
	≥60	6998	1711	51,397.0	33.3	5.19 (4.93, 5.46)	3.47 (3.29, 3.66)	2.83 (2.68, 2.98)	2.80 (2.65, 2.95)
	<30	151,978	5841	1,248,122.6	4.7	1 (Ref.)	1 (Ref.)	1 (Ref.)	1 (Ref.)
	<60	26,323	3661	206,206.0	17.8	3.83 (3.67, 3.99)	3.01 (2.89, 3.14)	2.72 (2.61, 2.84)	2.72 (2.6, 2.83)
	≥60	6998	1711	51,397.0	33.3	7.25 (6.87, 7.65)	4.83 (4.57, 5.10)	4.06 (3.84, 4.30)	4.05 (3.82, 4.29)
	*p* for trend					<0.001	<0.001	<0.001	<0.001

IR *: Incident rate (per 1000 person years); ^§^ Model 1: not adjusted; Model 2: adjusted for age, income, smoking status, alcohol intake, level of physical activity, and fasting glucose level; ^¥^ Model 3: further adjusted for hypertension, dyslipidemia, and chronic kidney disease.

## Data Availability

Data use was approved by the Korean National Health Insurance Corporation, and the results do not necessarily represent their opinion.
